# Comparison of the Pharmacological Effects of Paricalcitol and Doxercalciferol on the Factors Involved in Mineral Homeostasis

**DOI:** 10.1155/2010/621687

**Published:** 2010-03-02

**Authors:** J. Ruth Wu-Wong, Masaki Nakane, Gerard D. Gagne, Kristin A. Brooks, William T. Noonan

**Affiliations:** ^1^Department of Pharmacy Practice, University of Illinois at Chicago, Chicago, IL 60612-7230, USA; ^2^VidaGene, VDR Project, Chicago, IL 60612, USA; ^3^Abbott Laboratories, Renal Care, Abbott Park, IL 60048, USA

## Abstract

Vitamin D receptor agonists (VDRAs) directly suppress parathyroid hormone (PTH) mRNA expression. Different VDRAs are known to have differential effects on serum calcium (Ca), which may also affect serum PTH levels since serum Ca regulates PTH secretion mediated by the Ca-sensing receptor (CaSR). In this study, we compared the effects of paricalcitol and doxercalciferol on regulating serum Ca and PTH, and also the expression of PTH, VDR, and CaSR mRNA. The 5/6 nephrectomized (NX) Sprague-Dawley rats on a normal or hyperphosphatemia-inducing diet were treated with vehicle, paricalcitol, or doxercalciferol for two weeks. Both drugs at the tested doses (0.042–0.33 *μ*g/kg) suppressed PTH mRNA expression and serum PTH effectively in the 5/6 NX rats, but paricalcitol was less potent in raising serum Ca than doxercalciferol. In pig parathyroid cells, paricalcitol and the active form of doxercalciferol induced VDR translocation from the cytoplasm into the nucleus, suppressed PTH mRNA expression and inhibited cell proliferation in a similar manner, although paricalcitol induced the expression of CaSR mRNA more effectively. The multiple effects of VDRAs on modulating serum Ca, parathyroid cell proliferation, and the expression of CaSR and PTH mRNA reflect the complex involvement of the vitamin D axis in regulating the mineral homeostasis system.

## 1. Introduction

The steroid hormone, 1,  25-dihydroxyvitamin D_3_  (1, 25(OH)_2_D_3_, calcitriol), activates multiple signaling pathways in various cells and tissues. Although the synthesis of vitamin D_3_ occurs naturally in the skin with adequate sunlight exposure, vitamin D_3_ is not active and needs to be converted to 25(OH)D_3_ in the liver. From the liver, 25(OH)D_3_ is transported to the kidney and hydroxylated by 25-hydroxyvitamin D_3_ 1*α*-hydroxylase to form the active hormone, 1, 25-dihydroxyvitamin D_3_ or calcitriol [1]. Calcitriol is metabolized by 25-hydroxyvitamin D-24-hydroxylase (CYP24A1) [2] to yield the biliary excretory product calcitroic acid. The binding of 1, 25-dihydroxyvitamin D_3_ or its analogs to the vitamin D receptor (VDR), a nuclear receptor, activates VDR to recruit cofactors to form the VDR/cofactor complex, which binds to vitamin D response elements in the promoter region of target genes to regulate gene transcription [3].

During the past three decades, a majority of the studies in the VDR field have focused on elucidating its role in mineral homeostasis, which covers regulation of parathyroid hormone, intestinal calcium, and phosphate absorption and bone metabolism [4]. As a result of those studies, many new VDR agonists or activators (VDRAs) have been developed in an effort to curtail the increases in serum Ca associated with calcitriol. Currently new VDRAs such as paricalcitol and doxercalciferol are commonly used to manage hyperparathyroidism secondary to chronic kidney disease (CKD) [1]. Paricalcitol, like calcitriol, activates VDR directly, while doxercalciferol is inactive until it is metabolized in the liver to form 1, 25(OH)_2_D_2 _ (the major active metabolite) and 1, 24(OH)_2_D_2 _(the minor active metabolite). 

The serum PTH level is maintained by various mechanisms. Decreases in serum calcium (Ca) (hypocalcemia) and prolonged increases in serum phosphate (hyperphosphatemia) stimulate the parathyroid gland to secrete PTH from its storage granules. Ca regulates PTH secretion from the storage mediated by the Ca-sensing receptor (CaSR), while VDRAs down-regulate PTH gene expression at the transcriptional level. However, different VDRAs also exert differential effects on raising serum Ca, which may affect serum PTH levels. In an effort to investigate how the vitamin D axis modulates different factors involved in the mineral homeostasis system, we compared two VDRAs, paricalcitol and doxercalciferol, on modulating serum Ca and PTH, and the expression of VDR, PTH and CaSR mRNA in 5/6 nephrectomized rats and in primary culture of pig parathyroid cells. We also studied the effect of VDRAs on parathyroid cell proliferation and employed confocal microscopy to examine the VDR subcellular distribution pattern after parathyroid cells were treated with paricalcitol or active doxercalciferol. Our results suggest that paricalcitol and doxercalciferol regulate multiple factors involved in the mineral homeostasis system in a similar manner with some subtle differences.

## 2. Methods

### 2.1. Materials

1*α*-hydroxyvitamin D_2_ (1*α*(OH)D_2_, doxercalciferol), the major active metabolite of doxercalciferol (1*α*, 25-dihydroxyvitamin D_2_, 1, 25(OH)_2_D_2_), and 19-nor-1*α*, 25-dihydroxyvitamin D_2_ (19-nor-1*α*, 25-(OH)_2_D_2_, paricalcitol) were from Abbott Laboratories. Other reagents were of analytical grade. 

### 2.2. Subtotally Nephrectomized Rats

The 5/6 nephrectomized (NX) uremic rats were obtained from Charles River. The nephrectomy was performed on male, Sprague-Dawley rats with a standard two-step surgical ablation procedure. About six weeks after the surgery, the rats were treated with vehicle (5% ethanol +95% propylene glycol, 0.4 mL/kg), paricalcitol or doxercalciferol at the indicated doses intraperitoneally (i.p.), 3 times/week, for two weeks. Twenty-four hours after the last dosing, animals were anesthetized with ketamine and blood samples were collected. In the study measuring PTH mRNA in the parathyroid gland, about two weeks after the surgery the rats were put on a hyperphosphatemia-inducing diet containing 0.9% phosphorous and 0.6% calcium for 4 weeks, followed by treatment with vehicle, paricalcitol or doxercalciferol at the indicated dose (i.p., 3 times/week) for two weeks. Twenty-four hours after the last dosing, animals were anesthetized with ketamine and blood and parathyroid gland were collected.

### 2.3. Measurements of Physiological Parameters

Serum total calcium (Ca), serum phosphorus (Pi), creatinine, and BUN concentrations were measured using an Abbott Aeroset. Serum PTH was measured using a rat intact parathyroid hormone (PTH) ELISA kit obtained from ALPCO (Windham, NH). Blood iCa was determined using an i-STAT portable clinical analyzer with an ^EG^7+ cartridge. 

### 2.4. Real-Time RT-PCR

Real-time reverse Transcription-PCR was performed with a iCycler (BioRad, Hercules, CA). Each sample has a final volume of 25 *μ*L containing 100 ng of cDNA, 0.4 *μ*M each of the forward and reverse PCR primers and 0.1 *μ*M of the TaqMan probe (Applied Biosystems). Temperature conditions consisted of a step of 5 minutes at 95°C, followed by 40 cycles of 60°C for 1 minute and 95°C for 15 seconds. Data was collected during each extension phase of the PCR reaction and analyzed with the software package (BioRad). Threshold cycles were determined for each gene. 

### 2.5. Pig Parathyroid Cell Cultures

Freshly harvested pig parathyroid glands were purchased from Analytical Biological Systems (Wilmington, DE). Dispersed pig parathyroid cells were prepared using a method adapted from those previously described for isolating bovine parathyroid cells [5, 6]. Briefly, pig parathyroid glands were rinsed in 100% ethanol first and then in ice-cold Buffer A (20 mM HEPES, pH 7.5, in DMEM without bicarbonate, plus 1 mM Ca, 1 mM magnesium, 500 units/mL penicillin, 500 *μ*g/mL streptomycin, 1.25 *μ*g/mL amphotericin B, and 100 *μ*g/mL gentamycin). The glands were trimmed of extraneous fatty tissue and finely minced into fragments. The minced pieces were washed twice with ice-cold PBS and incubated in Buffer A containing 2 mg/mL collagenase Type 1 (Worthington no. 4196) and 50 *μ*g/mL DNAse 1 (Sigma no. DN25, 10 mL/gram of tissue) for 1.5–3.0 hours at 37°C in a CO_2_ incubator. During incubation the tissue was stirred every 30 minutes. At the end of the digestion period large pieces were removed and the cell suspension was filtered through 70 *μ*m and then 40 *μ*m cell strainers (BD, Falcon). The cells were washed 3X with ice-cold PBS and gently resuspended in Medium A containing DMEM: Ham's F-12 (50  :  50), 10% FBS, 1.2 mM Ca, 15 mM HEPES, 100 IU/mL penicillin, 100 *μ*g/mL streptomycin, 0.25 *μ*g/mL amphotericin B, 1 : 100 Insulin-Tranferrin-Selenium (GIBCO). The cells were then incubated overnight at 37°C in a CO_2_ incubator, and washed for at least 3 times in Medium A. A > 80% viability (as determined by trypan blue staining) was obtained. Cells were used within 2 days for experiments.

### 2.6. Proliferation Assay

Pig parathyroid cells were plated at 1 × 10^7^ cells/well into 48-well plates (Corning, Corning, NY). The cell number was determined at plating (Day 0) and after cells were treated with test agents for 72 hours. 

### 2.7. Confocal Microscopy

Cells grown in four-chamber slides were treated with test agent for different periods of time. Cells were washed with PBS for 30 seconds, fixed with 4% formaldehyde in PBS for 15 minutes, washed again with PBS, and then treated with 0.2% Triton X-100 in PBS for 5 minutes. The slides were rinsed with PBS and incubated with PBS plus 1% BSA for 1 hour at room temperature. The slides were then incubated with a mouse anti-VDR monoclonal antibody (50-fold dilution, Santa Cruz Biotechnology, Santa Cruz, CA) in PBS for >20 hours at 4°C. After incubation, slides were rinsed with PBS, blocked with 1% BSA in PBS for 15 minutes, and then incubated with a secondary antibody (Alexa Fluor 488 goat antimouse IgG; Molecular Probes, Eugene, OR) for 1 hour at 37°C, followed by another wash with PBS. To stain nuclei, slides were treated with ribonuclease-A (Sigma, St. Louis, MO) at 100 *μ*g/mL in PBS for 20 minutes at 37°C, followed by PBS wash and then incubated with propidium iodide (10 *μ*g/mL, Molecular Probes) for 15 minutes at room temperature and washed again with PBS. The slides were mounted and photographed with a Bio-Rad MRC 1024 confocal microscope.

## 3. Results

**Figure 1:**
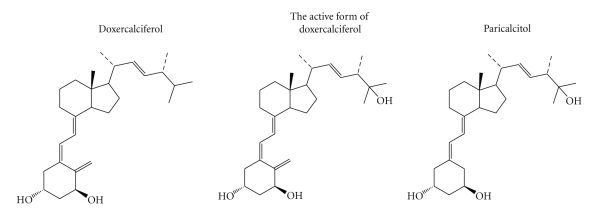
The structures of doxercalciferol, the active form of doxercalciferol and paricalcitol.

**Figure 2:**
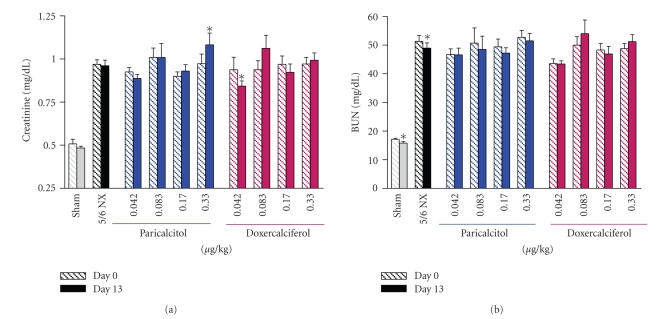
The serum creatinine and BUN levels in 5/6 nephrectomized (NX) uremic rats. 5/6 NX rats on normal diet were treated with vehicle, paricalcitol, or doxercalciferol at indicated doses, i.p., 3 times/week, for two weeks (*n* = 8–12 per group). Sham-vehicle rats were dosed with vehicle. Handling of animals was as described in Materials and Methods. Blood samples were collected for the measurement of serum creatinine and BUN. Mean ± standard error was calculated for each group. Unpaired t-test with 95% confidence intervals of difference was performed for statistical comparisons. *P < .05 versus Day 0 (before dosing at six weeks after the surgery). Day 13: after dosing with vehicle or drugs.

**Figure 3:**
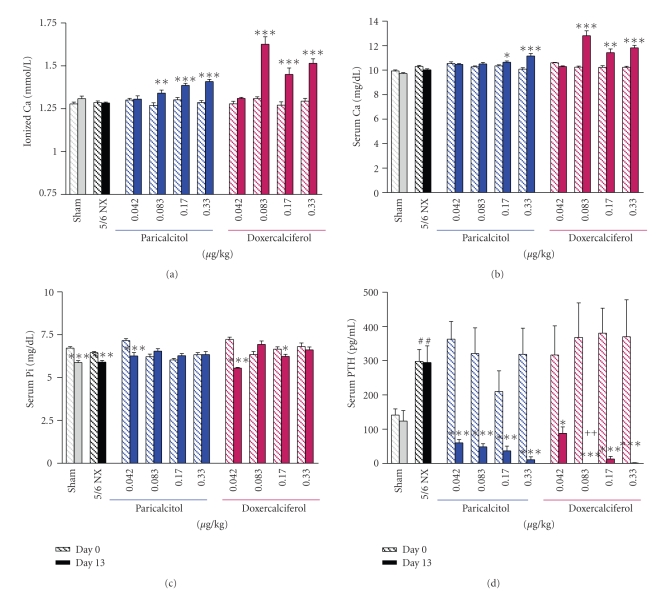
The ionized Ca, serum Ca, Pi, and PTH levels in uremic rats. Rats were treated as in Figure 2. Blood samples were collected for the measurement of ionized Ca, serum Ca, Pi, and PTH levels. Mean ± standard error was calculated for each group. Unpaired t-test with 95% confidence intervals of difference was performed for statistical comparisons. *P < .05, **P < .01, ***P < .001 versus Day 0 (before dosing at six weeks after the surgery). ^#^ *P* < .05 versus Sham. ^++^ *P* < .01 versus paricalcitol at the same dose.

The structures of doxercalciferol, the major active metabolite of doxercalciferol (1, 25(OH)_2_D_2_), and paricalcitol are shown in Figure 1. The effects of paricalcitol and doxercalciferol on physiological parameters were first compared in the 5/6 NX rats on a normal diet. Figure 2 show that the serum creatinine and BUN levels were significantly elevated in the 5/6 nephrectomized (NX) rats (versus Sham-vehicle), indicating a uniform disease state. Paricalcitol or doxercalciferol at the tested doses had no dose-dependent effect on creatinine or BUN (versus 5/6 NX-vehicle). Figures 3(a) and 3(b) shows that both ionized Ca and serum Ca were not significantly changed in the 5/6 NX-vehicle group (versus Sham). Paricalcitol and doxercalciferol induced an increase in serum and ionized Ca in a dose-dependent manner; the effect of doxercalciferol was much more profound than that of paricalcitol. Although there was variation in the serum phosphate (Pi) values (Figure 3(c)), both drugs at the tested doses did not have a significant effect. These results are consistent with a previous report by Slatopolsky et al. [7] that paricalcitol is less hypercalcemic than doxercalciferol. Figure 3(d) shows that serum PTH was elevated in the 5/6 NX rat, and was significantly reduced by either paricalcitol or doxercalciferol at the tested doses.

**Figure 4:**
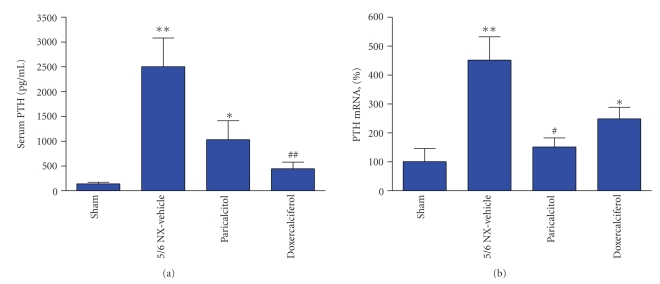
Effects of paricalcitol and doxercalciferol on serum PTH and PTH mRNA levels in uremic rats. The 5/6 NX rats on a hyperphosphatemia-inducing diet were treated with vehicle, paricalcitol, or doxercalciferol at 0.33 *μ*g/kg, i.p., 3 times/week, for two weeks (*n* = 8–11 per group). Blood samples and parathyroid gland were collected for the measurement of serum PTH (a) and PTH mRNA (b). For (b), real-time RT-PCR was performed as described in Materials and Methods. The mRNA expression level was first normalized to the GAPDH mRNA level and then calculated as % of control (Sham-vehicle, 100%). One way ANOVA Dunnett test with 95% confidence intervals of difference was performed for statistical comparisons. *P < .05, **P < .01 versus Sham. ^#^ *P* < .05, ^##^ *P* < .01 versus 5/6 NX-Vehicle.

We then determined the effects of these two drugs on PTH mRNA expression in the 5/6 NX rats. In order to induce parathyroid gland hypertrophy so that enough tissues could be collected for real-time PCR analysis, the 5/6 NX rats were put on a hyperphosphatemia-inducing diet containing 0.9% phosphorous and 0.6% calcium [8] for 4 weeks, followed by treatment with vehicle or drugs for two weeks. Table 1 summarizes the physiological parameters. Similar to the study in Figures 2 and 3, the serum creatinine and BUN levels were significantly elevated in the 5/6 NX rats (versus Sham). Paricalcitol at or doxercalciferol at 0.33 *μ*g/kg had no significant effect on creatinine or BUN. The serum Ca or iCa level was lower in the NX-vehicle group, while serum Pi was significantly higher. Both drugs at the tested dose raised serum Ca and iCa, but did not significantly change the serum phosphate level (versus NX-vehicle). The CaxPi product was increased by the drug treatment, although the value in the paricalcitol group failed to reach a statistical significance (versus NX-vehicle). Figure 4 shows that the combination of 5/6 nephrectomy and the hyperphosphatemia-inducing diet caused a 17-fold increase in the serum PTH level and a 4.6-fold increase in PTH mRNA (versus Sham). Both paricalcitol and doxercalciferol at the tested dose reduced serum PTH and PTH mRNA.

**Figure 5:**
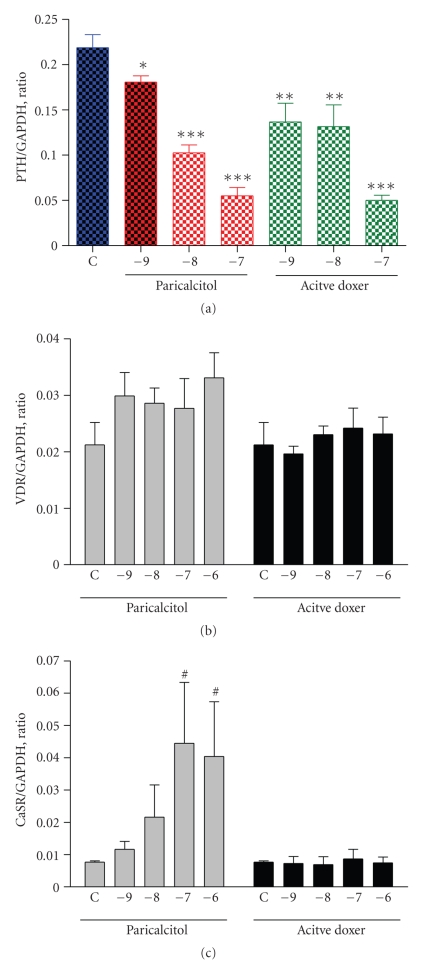
Effect of paricalcitol and active doxercalciferol on the expression of PTH, VDR, and CaSR mRNA in pig parathyroid cells. Cells were treated with paricalcitol and active doxercalciferol at indicated concentrations (in log *M*) for 24 hours. Samples were processed and real-time RT-PCR performed. The mRNA expression level was normalized to the GAPDH mRNA level. C: control (no drug treatment); doxer: doxercalciferol. Values shown are mean ± the standard deviation (*n* = 4). One way ANOVA Dunnett test with 95% confidence intervals of difference was performed for statistical comparisons. *P < .05, **P < .01, ***P < .001 versus Control (C, no drug treatment). ^#^ *P* < .05 versus Control (C).

To further investigate the effects of paricalcitol and doxercalciferol on PTH mRNA expression, we treated primary culture of pig parathyroid cells with different concentrations of paricalcitol or the major active metabolite of doxercalciferol (1*α*, 25-(OH)_2_D_2_, active doxercalciferol). Figure 5(a) shows the results from real-time RT-PCR analysis that paricalcitol decreased PTH mRNA effectively in a dose-dependent manner achieving a 75% inhibition at 100 nM. Active doxercalciferol also suppressed PTH mRNA in a similar manner. Figure 5(b) shows that paricalcitol or active doxercalciferol did not have a significant effect on VDR mRNA expression. Figure 5(c) shows that paricalcitol induced the expression of CaSR mRNA in a dose-dependent manner, while active doxercalciferol had no effect. 

**Figure 6:**
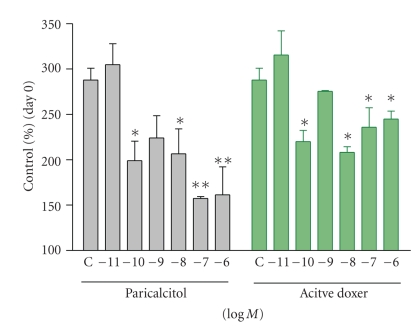
Effect of paricalcitol and active doxercalciferol on the proliferation of pig parathyroid cells. Cells were treated with or without increasing concentrations of paricalcitol or active doxercalciferol (doxer) for 72 hours. Data were expressed as % of control (cells on Day 0, 100%). Each value shown is mean ± the standard deviation (*n* = 4–8). One way ANOVA Dunnett test with 95% confidence intervals of difference was performed for statistical comparisons. *P < .05, **P < .01 versus Control (C, no drug treatment).

Figure 6 compares the effects of paricalcitol and active doxercalciferol on the proliferation of the pig parathyroid cells. During the 72 hours incubation period, the number of parathyroid cells increased by ~3-fold (versus Day 0). Both drugs inhibited the proliferation of these cells. 

**Figure 7:**
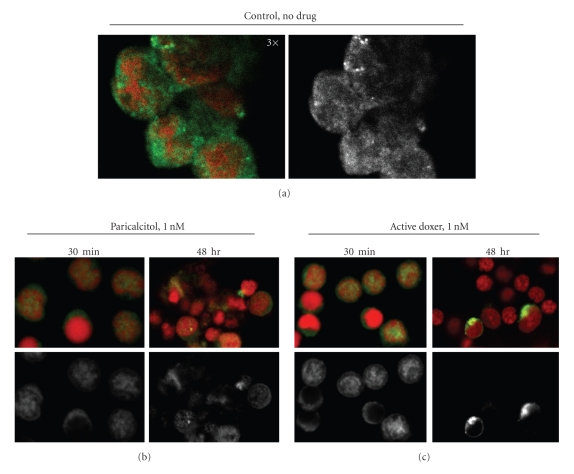
Localization of VDR in pig parathyroid cells. Cells were incubated without (a) or with 1 nM paricalcitol (b) or active doxercalciferol (c) for 30 minutes or 48 hours, and then stained for VDR (green) and nucleus (red). The VDR staining alone was also shown in black and white. (a): 3× magnification; (b) and (c): 2× magnification; doxer: doxercalciferol.

We then treated the parathyroid cells with 1 nM paricalcitol or active doxercalciferol for 30 minutes or 48 hours, and then fixed and stained cells with an anti-VDR antibody to examine the effect of these two drugs on the subcellular localization of VDR. The nuclei were stained by propidium iodide (red color). Figure 7 shows representative fields from confocal microscopy. The pig parathyroid cells contained a thin layer of cytoplasm over a large nucleus. In the absence of VDRAs, VDR staining (green color) seemed more dispersed in the cytoplasm in ~90% of cells; approximately 10% of cells exhibited strong VDR staining in the nuclei (Figure 7(a)). Figures 7(b) and 7(c) show that, upon treatment with 1 nM paricalcitol or active doxercalciferol for 30 minutes, an increase in VDR localization in the nucleus was observed in >80% of cells; the effects of both drugs were similar. Interestingly, in cells treated with 1 nM paricalcitol continuously for 48 hours, VDR localization was still observed in the nucleus in >60% of the cells. As a comparison, >80% of the cells treated with 1 nM active doxercalciferol for 48 hours exhibited no VDR staining, and the remaining VDR-positive cells exhibited a bright ring of green color (VDR staining) surrounding the nucleus.

## 4. Discussion

VDRAs regulate PTH at the transcriptional level. However, the serum PTH level is maintained by other mechanisms including serum Ca and phosphate, which modulates the parathyroid gland to secrete PTH from its storage granules. It is known that different VDRAs such as calcitriol, doxercalciferol, and paricalcitol exhibit different effects on raising serum Ca. Calcitriol is about 10-fold more hypercalcemic than paricalcitol, while doxercalciferol is ~2-3-fold more hypercalcemic [2, 9–11]. To investigate whether the different hypercalcemic effect of VDRAs plays a role in serum PTH suppression, we studied paricalcitol and doxercalciferol because these two drugs are similar in potency in suppressing serum PTH with different hypercalcemic effects. Our data from the 5/6 NX rats are consistent with the previous studies [9–11] that paricalcitol and doxercalciferol are equally efficacious in suppressing serum PTH, but paricalcitol is less hypercalcemic than doxercalciferol.

Since it is not practical to isolate parathyroid cells from the rat due to the limited availability of the tissue, we prepared primary culture of pig parathyroid cells. Previously it has been shown that, in dispersed bovine parathyroid cells, calcitriol reduced the expression of PTH [12]. Our results using the pig parathyroid cells are consistent that VDRAs suppress PTH mRNA expression and inhibit parathyroid cell proliferation. Paricalcitol and active doxercalciferol have no significant effects on modulating the VDR mRNA level in the parathyroid cells, although paricalcitol seems more effective in inducing the expression of CaSR mRNA. Paricalcitol may “prime” the parathyroid cells to become more sensitive to serum Ca since paricalcitol is less effective in raising serum Ca levels. 

We demonstrate by confocal microscopy that VDRA treatment of the pig parathyroid cells increases VDR staining in the nucleus, which can be seen as early as 30 minutes after the addition of paricalcitol or active doxercalciferol. The observation, consistent with our previous studies in the HL-60 promyelocytic leukemia cells, demonstrates that VDR resides predominantly in the cytoplasm of the pig parathyroid cells in the absence of VDRAs, and VDRA treatment induces VDR to translocate into the nucleus [13]. Our observations seem to suggest that different VDRAs may have differential effects on the subcellular distribution of VDR after a prolonged incubation period. It is well known that binding of agonists to the receptor stabilizes the VDR via protecting the receptor from proteolytic degradation. Therefore, it is possible that the different effects of these two compounds on nuclear VDR staining could be an indication of a differential intraparathyroid catabolism of the ligand-bound VDR. 

From this study, we demonstrated that the effect of VDRAs on suppressing serum PTH involves multiple factors such as regulation of PTH and CaSR mRNA expression, inhibition of parathyroid cell proliferation, and modulation of serum Ca. These observations reflect the complexity of the vitamin D axis in regulating the mineral homeostasis system.

## Figures and Tables

**Table 1:** Physiological parameters in Sham rats or 5/6 nephrectomized rats fed a hyperphosphatemia-inducing diet.

Parameters	Sham	5/6 NX-vehicle	5/6 NX-paricalcitol	5/6 NX-doxercalciferol
	—	—	0.33 *μ*g/kg	0.33 *μ*g/kg
Creatinine (mg/dL)	0.45 ± 0.03	1.28 ± 0.13***	1.57 ± 0.33**	1.29 ± 0.11***
BUN (mg/dL)	12.3 ± 0.3	59.1 ± 5.3***	60.1 ± 9.0***	60.5 ± 5.0***
Ionized Ca (mmil/L)	1.35 ± 0.02	1.13 ± 0.04**	1.26 ± 0.06^#^	1.35 ± 0.03^###^
Total serum Ca (mg/dL)	9.95 ± 0.13	8.99 ± 0.35**	10.1 ± 0.27^#^	10.93 ± 0.23^∗∗###^
Total serum Pi (mg/dL)	6.50 ± 0.25	10.09 ± 0.76**	12.05 ± 1.89*	11.31 ± 1.05**
CaxPi ((mg/dL)^2^)	64.8 ± 3.3	89.1 ± 4.0*	120.3 ± 18.2*	122.9 ± 10.6^∗∗∗###^
Serum PTH	142 ± 31	2504 ± 579**	1031 ± 382*	445 ± 135^##^

Mean ± SEM; *P < .05, **P < .01, ***P < .001 versus Sham; ^#^ *P* < .05, ^##^ *P* < .01, ^###^ *P* < .001 versus NX-vehicle; *n* = 8–11 per group.

## Abbreviations

VDR: Vitamin D receptor
PTH: Parathyroid hormone
NX: 5/6 nephrectomized
CKD: Chronic kidney disease.
